# Genome Sequence of *Pasteurella multocida* Strain Razi_Pm0001

**DOI:** 10.1128/genomeA.01532-16

**Published:** 2017-02-02

**Authors:** Lisa D. Sprague, Keyvan Tadayon

**Affiliations:** a Friedrich-Loeffler-Institut, Federal Research Institute for Animal Health, Institute for Bacterial Infections and Zoonoses, Jena, Germany; b Department of Microbiology, Razi Institute, Agriculture Research, Education and Extension Organization, Karaj, Iran

## Abstract

We report here the genome sequence of *Pasteurella multocida* Razi_Pm0001 from bovine origin, isolated in Iran in 1936. The genome has a size of 2,360,663 bp, a G+C content of 40.4%, and is predicted to contain 2,052 coding sequences.

## GENOME ANNOUNCEMENT

In 1936, a strain of *Pasteurella  multocida* was isolated from diseased cattle and used for the preparation of a hemorrhagic septicemia (HS) vaccine at the Razi Institute in Iran (1). This article announces the *de novo* genome sequence of *P. multocida* Razi_Pm0001.

 Genomic DNA was isolated from an ethanol-inactivated overnight culture grown in triptose phosphate broth with a Qiagen genomic tip 100/Q and genomic DNA buffer set (Qiagen, Hilden, Germany). DNA quality was examined by using both a NanoDrop spectrophotometer (Thermo Scientific, Schwerte, Germany) and a Qubit version 2.0 fluorometer (Life Technologies, Inc., Darmstadt, Germany). Whole-genome sequencing was performed on the Pacific Biosciences RS sequencer, with SMRT Technology PacBio RS II (Pacific Biosciences, Menlo Park, CA, USA), at GATC (Konstanz, Germany), using standard protocols according to the manufacturer’s instructions, which were followed throughout the sequencing process (2). *De novo* assembly was performed with SMRT portal version 2.3.0 (Daemon version 2.3.0, SMRT View version 2.3, and SMRTpipe version 2.3.0; Pacific Biosciences) from one contig, with an *N*_50 _contig length of 2,360,663 bp. The genome has a size of 2,360,663 bp and a G+C content of 40.5% (Geneious 9.0.5; Biomatters, Auckland, New Zealand). Genome annotation was done using the NCBI Prokaryotic Genome Annotation Pipeline (http://www.ncbi.nlm.nih.gov/genome/annotation_prok) and predicted 2,222 genes, 88 pseudogenes, 2,052 coding sequences, 59 tRNAs, seven 5S rRNAs, six 16S rRNAs, six 23S rRNAs, and four ncRNAs.

### Accession number(s).

The sequence of *P. multocida* Razi_Pm0001 has been deposited at DDBJ/EMBL/GenBank under the accession number CP017961.

## References

[1] Delpy LP, Rastegar R 1938 Sur une nouvelle méthode de vaccination contre la pasteurellose des bovins et de buffles. Rev Immunol 4:322–339.

[2] Eid J, Fehr A, Gray J, Luong K, Lyle J, Otto G, Peluso P, Rank D, Baybayan P, Bettman B, Bibillo A, Bjornson K, Chaudhuri B, Christians F, Cicero R, Clark S, Dalal R, Dewinter A, Dixon J, Foquet M, Gaertner A, Hardenbol P, Heiner C, Hester K, Holden D, Kearns G, Kong X, Kuse R, Lacroix Y, Lin S, Lundquist P, Ma C, Marks P, Maxham M, Murphy D, Park I, Pham T, Phillips M, Roy J, Sebra R, Shen G, Sorenson J, Tomaney A, Travers K, Trulson M, Vieceli J, Wegener J, Wu D, Yang A, Zaccarin D, Zhao P, Zhong F, Korlach J, Turner S 2009 Real-time DNA sequencing from single polymerase molecules. Science 323:133–138. doi:10.1126/science.1162986. 19023044

